# Multiple Roles of HIV-1 Capsid during the Virus Replication Cycle

**DOI:** 10.1007/s12250-019-00095-3

**Published:** 2019-04-26

**Authors:** Mariia Novikova, Yulan Zhang, Eric O. Freed, Ke Peng

**Affiliations:** 1 Virus-Cell Interaction Section, HIV Dynamics and Replication Program, Center for Cancer Research, National Cancer Institute, Frederick, MD 21702 USA; 2 State Key Laboratory of Virology, Wuhan Institute of Virology, Chinese Academy of Sciences, Wuhan, 430071 China

**Keywords:** Human immunodeficiency virus-1 (HIV-1), Capsid (CA), Assembly, Post entry, Uncoating and nuclear import, Inhibitor

## Abstract

Human immunodeficiency virus-1 capsid (HIV-1 CA) is involved in different stages of the viral replication cycle. During virion assembly, CA drives the formation of the hexameric lattice in immature viral particles, while in mature virions CA monomers assemble in cone-shaped cores surrounding the viral RNA genome and associated proteins. In addition to its functions in late stages of the viral replication cycle, CA plays key roles in a number of processes during early phases of HIV-1 infection including trafficking, uncoating, recognition by host cellular proteins and nuclear import of the viral pre-integration complex. As a result of efficient cooperation of CA with other viral and cellular proteins, integration of the viral genetic material into the host genome, which is an essential step for productive viral infection, successfully occurs. In this review, we will summarize available data on CA functions in HIV-1 replication, describing in detail its roles in late and early phases of the viral replication cycle.

## Introduction

**Fig. 1:**
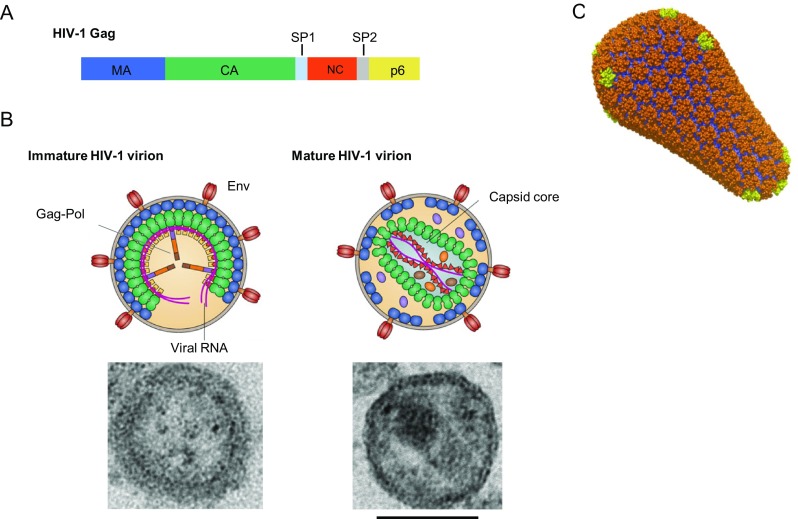
Structure of the HIV-1 virion and its components. **A** HIV-1 Gag domain organization. The HIV-1 Gag consists of four domains—matrix (MA), capsid (CA), nucleocapsid (NC) and p6—and two short peptides, SP1 and SP2, that are cleaved into distinct proteins by the viral protease during maturation. **B** Immature and mature HIV-1 virions. In the immature virion (left) Gag molecules are radially organized in the hexameric lattice (Gag domains are shown in the same colors as in **A**). Two molecules of viral genomic RNA (magenta) per virion are packaged. Trimers of Env protein are embedded in the viral membrane. Gag-Pol molecules that produce viral enzymes—protease (PR) (purple), reverse transcriptase (RT) (orange) and integrase (IN) (brown)—are present in 1:20 ratio to Gag. In the mature virion (right), MA retains bound to the viral membrane, released CA forms a characteristic cone-shaped core, NC is bound to the viral genomic RNA (reprinted from Freed 2015). Electron microscopy images of the immature (left) and mature (right) virions are shown (Novikova and Freed, unpublished data). Scale bar, 100 nm. **C** Structure of the mature capsid (Pornillos *et al.*^2011^), reprinted with permission from Nature Publishing Group. Capsid is formed by a hexameric CA lattice (CA-NTDs in orange, CA-CTDs in blue) with 12 embedded CA pentamers (yellow) allowing for the closing off of both ends of the conical structure.

Assembly and release of HIV-1 virions occur predominantly at the plasma membrane of infected cells (Sundquist and Krausslich 2012; Freed 2015). The major structural viral protein, Gag, is essential and sufficient for the formation of virus-like particles (VLPs). Gag is synthesized in the cytosol as a polyprotein composed of four domains—matrix (MA), capsid (CA), nucleocapsid (NC) and p6—and two short peptides, SP1 and SP2 (Fig. 1A). An N-terminal myristyl group, and a cluster of basic residues in MA, that binds the phospholipid PI(4,5)P_2_, appear to be the main determinants of Gag targeting and binding to the inner leaflet of the plasma membrane (Ono *et al.*^2004^; Tang *et al.*^2004^; Saad *et al.*^2006^). CA–CA interactions play a central role in driving the formation of a hexameric Gag lattice that contains gaps in the released virion to form a spherical shape (Wright *et al.*^2007^; Carlson *et al.*^2008^; Briggs *et al.*^2009^). NC is responsible for packaging of the viral genomic RNA, and p6 is required for budding of newly assembled virions. For conversion into a fully infectious particle, incorporation of the Gag-Pol polyprotein comprising three viral enzymes—reverse transcriptase (RT), integrase (IN) and protease (PR)—is needed. Concomitant with or shortly after budding of the immature virion, PR cleaves the Gag precursor molecules into the distinct proteins thus initiating the maturation process (Fig. 1B). These PR-driven proteolysis events result in significant structural rearrangements of the virion interior, including the assembly of the fullerene-like, cone-shaped core, which is essential for productive viral infection. The core is composed of the mature capsid, a protein shell formed by ~ 250 CA hexamers and 12 CA pentamers that close off both ends of the structure, and the viral ribonucleoprotein complex (Ganser *et al.*^1999^; Pornillos *et al.*^2011^) (Fig. 1C). In the cytoplasm of a newly infected cell the reverse transcription of the viral genomic RNA results in the formation of a double stranded viral DNA that is transported to the nucleus and integrates into the host cell chromatin. It has been shown that CA plays an active role in many events early post-infection, prior to viral DNA integration (Fassati 2012; Campbell and Hope 2015; Yamashita and Engelman 2017). The integrated viral genome (the “provirus”) is transcribed by the host transcription machinery followed by translation of viral transcripts into viral proteins needed for assembly of new HIV-1 particles.

In the first part of this review we summarize the current data on the structural role of CA in assembly of HIV-1 virions. In addition, CA-binding inhibitors of late stages of the viral replication cycle will be reviewed. In the second part, we will focus on CA-mediated processes occurring after fusion of the HIV-1 virion with the target cell and before viral DNA integration in the host genome. Host restriction factors that counteract CA functions will be also briefly discussed.

## Role of CA in HIV-1 Assembly and Maturation

### Structure of the CA Monomer

**Fig. 2:**
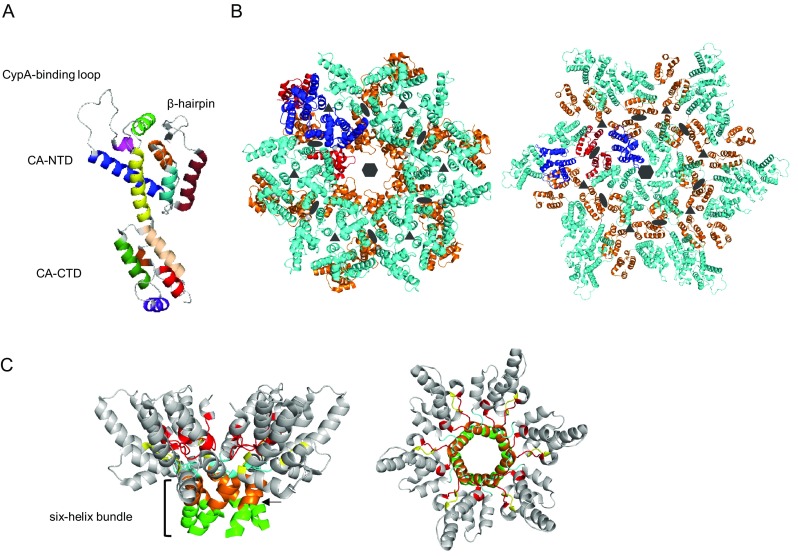
Arrangement of CA in immature and mature HIV-1 virions. **A** Structure of the CA monomer. CA consists of two α-helical domains—CA-NTD and CA-CTD—that are connected by a short linker. The CypA-binding loop and β-hairpin are indicated. Helices of CA-NTD: helix 1 (ruby), helix 2 (cyan), helix 3 (orange), helix 4 (blue), helix 5 (magenta), helix 6 (light green) and helix 7 (yellow). Helices of CA-CTD: 3_10_ helix (brown), helix 8 (wheat), helix 9 (green), helix 10 (purple) and helix 11 (red) [PDB ID: 5MCX (Mattei *et al.*2016a)]. **B** Structural arrangement of the CA layer in intact immature (left) and mature (right) HIV-1 virions. CA-NTDs and CA-CTDs of two CA monomers from neighbouring hexamers are colored in blue and red, respectively. All other CA-NTDs and CA-CTDs are in cyan and in orange, respectively. Some important interfaces involved in the formation of the two structures are shown. Hexagons indicate a sixfold interface in the individual hexamer in both structures. In the immature lattice (left) homo-dimeric (ovals) and homo-trimeric (triangles) interfaces important for connecting neighboring hexamers are formed by helices 1 and 2 of CA-NTDs, respectively [PDB ID: 4USN (Schur *et al.*^2015^)]. In the mature virion (right), inter-hexamer interactions are formed by helices 10 and 11 residing at threefold interfaces (triangles) and by residues from helix 9 at twofold interfaces (ovals) [PDB ID: 5MCX (Mattei *et al.*2016a)]. **C** CA-CTD and the N-terminal seven residues of SP1 (side view—left; top view—right) in the six-helix bundle. Residues of SP1 (light green), CA-CTD (orange), MHR loop (red), loop connecting helices 9 and 10 (cyan), and a β-turn (yellow) are key elements for the formation of the CA-SP1 bundle in the immature Gag lattice [PDB ID: 5I4T (Wagner *et al.*^2016^)]. The arrow indicates the cleavage site between CA and SP1.

As mentioned above, CA is critical for assembly of both immature and mature viral particles. HIV-1 CA is an α-helical protein composed by two domains—an N-terminal domain (CA-NTD) and a C-terminal domain (CA-CTD)—connected by a short linker (Fig. 2A). CA-NTD consists of seven α-helices and a characteristic extended cyclophilin A (CypA)-binding loop (Gamble *et al.*^1996^; Gitti *et al.*^1996^). In the immature Gag lattice, the N-terminal end of CA-NTD is unstructured and linked to the membrane-bound MA domain, while in the context of the mature CA monomer the proteolytically released N-terminus folds into a β-hairpin (von Schwedler *et al.*^1998^). CA-CTD contains a short 3_10_ helix, the major homology region (MHR)—a highly conserved element in all orthoretroviruses required for viral replication—and four α-helices (Gamble *et al.*^1997^). Like the N-terminus of the CA-NTD, the C-terminal residues of the CA-CTD undergo significant structural rearrangements during maturation. In immature virions, the C-terminus of the CA domain is part of the CA-SP1 junction that forms a six-helix bundle (Fig. 2B, left; Fig. 2C), a critical structural element in the Gag hexamer (Wright *et al.*^2007^). Upon proteolysis at the CA-SP1 junction, this region becomes disordered (Gamble *et al.*^1997^). Although the structure of the mature CA monomer is highly conserved across retroviruses, the arrangement of CA subunits in the immature and mature lattices, and the shape of mature cores, vary significantly among retroviruses (Zhang *et al.*^2015^; Mattei *et al.*2016b).

### The Role of CA in Assembly of the Immature HIV-1 Gag Lattice

Numerous structural and mutational studies have characterized the role of HIV-1 CA in the immature and mature Gag lattices and revealed substantial differences in CA–CA contacts required for assembly of the two structures (Fig. 2B, left and right, respectively) (Lingappa *et al.*^2014^; Mattei *et al.*2016b). In the immature lattice, each CA-NTD forms an extensive network of interactions with CA-NTDs from the same and neighbouring hexamers. Within hexamers, residues in helix 4 of each CA-NTD interact with residues between helices 5 and 6 in neighbouring CA-NTDs. Inter-hexameric contacts are mediated by helices 1 and 2, which form homo-dimeric and homo-trimeric interfaces, respectively (Schur *et al.*^2015^) (Fig. 2B, left). Recently, the short loop between helices 6 and 7, located at the threefold inter-hexamer interface, was shown to be important for assembly of the immature Gag lattice (Novikova *et al.*^2018^). Interestingly, deletion of the entire CA-NTD only modestly decreases production of VLPs, although the resulting particles are more heterogeneous in size relative to the WT (Borsetti *et al.*^1998^). This finding indicates a major role for the CA-CTD in assembly of immature virions. The CA-CTD, similarly to the CA-NTD, is involved in the formation of both intra- and inter-hexameric contacts. Helix 9 forms a homo-dimeric interface linking neighboring hexamers, while residues from the MHR and other regions are important for generating the Gag hexamer. In contrast to the mature capsid, there are no extensive intra-protomer CA-NTD-CA-CTD contacts (Schur *et al.*^2015^). One of the distinctive features of the immature HIV-1 Gag lattice is the formation of goblet-like structures within individual hexamers in which the cup is formed by the CA-CTD and the stem represents a six-helix bundle formed by the CA-SP1 junction region, as determined by cryo-ET and X-ray crystallography (Schur *et al.*^2016^; Wagner *et al.*^2016^) (Fig. 2C). The six-helix bundle is formed by eight C-terminal residues of the CA-CTD and the N-terminal seven residues of SP1. Several MHR residues, the loop connecting helices 9 and 10 and a β-turn, formed by four residues downstream of helix 11, are required to maintain this structure and stabilize the hexamer (Wagner *et al.*^2016^). Two recent studies (Dick *et al.*^2018^; Mallery *et al.*^2018^) demonstrated that the negatively charged small molecule inositol hexaphosphate (IP6) facilitates assembly of the six-helix bundle in the immature Gag lattice, and upon Gag proteolysis promotes the formation of the mature capsid. Two CA residues, Lys158 and Lys227, from the MHR and the CA-SP1 junction, respectively, are arranged in two positively charged rings that constitute the IP6 binding site in the Gag lattice (Dick *et al.*^2018^; Mallery *et al.*^2018^).

Apart from the structural role of the CA-SP1 six-helix bundle in Gag lattice formation, this element also modulates the maturation process by sequestering the CA-SP1 PR cleavage site. Two preceding cleavages between MA-CA and SP1-NC are essential to destabilize the structure of the six-helix bundle, thus exposing the CA-SP1 cleavage site (Fig. 2C) to PR to complete Gag processing (Schur *et al.*^2016^; Wagner *et al.*^2016^). The CA-SP1 bundle is inherently flexible and conformationally dynamic, allowing for a balance between the requirement for stability during assembly and flexibility to expose the CA-SP1 cleavage site during maturation (Schur *et al.*^2016^; Wagner *et al.*^2016^) and see below. A recent cryo-ET study using a panel of Gag cleavage site mutants provided data suggesting that destabilization of the CA-SP1 bundle is a key determinant in the process of structural maturation (Mattei *et al.*^2018^).

### The Role of CA in the Formation of the Mature Capsid

Upon completion of PR-mediated Gag processing, the newly liberated CA proteins assemble into the cone-shaped capsid within the virion. The mechanism of mature lattice formation is still incompletely understood. Most studies have supported a disassembly and *de novo* reassembly model in which the released CA monomers reassemble into the conical capsid (Briggs *et al.*^2006^; Keller *et al.*^2013^; Woodward *et al.*^2015^). Some studies, however, support a displacive transition model, in which the immature CA lattice transforms into the mature capsid without free CA monomers being released into the virion interior (Meng *et al.*^2012^; Frank *et al.*^2015^). The formation of the core through a combination of both mechanisms has also been suggested (Ning *et al.*^2016^).

The structures of CA hexamers and pentamers, and their arrangement in the mature lattice, have been determined in cryo-EM and X-ray crystallography studies using assembled mature CA tubes and WT or cross-linked CA hexameric lattices (Li *et al.*^2000^; Pornillos *et al.*^2009^, ^2011^; Zhao *et al.*^2013^; Gres *et al.*^2015^). Recently, the CA arrangement in the mature capsid within HIV-1 virions has been resolved by cryo-ET (Mattei *et al.*2016a). The structure of hexamers in the intact core appears to be similar to one of the previously described structures (Gres *et al.*^2015^). Intra-hexamer interactions are formed by CA-NTD–CA-NTD and CA-NTD–CA-CTD contacts between adjacent CA monomers. There is also a substantial cluster of intra-subunit CA-NTD–CA-CTD contacts in the mature lattice. At the sixfold symmetry axis interface of the mature hexamer, the residue Arg18 from helix 1 constitutes a selective channel for incoming nucleotides needed for reverse transcription (Jacques *et al.*^2016^). The same Arg ring has been recently shown to be involved in coordination of IP6 molecules that promote assembly of CA hexamers and regulate capsid stability (Dick *et al.*^2018^; Mallery *et al.*^2018^). Hexamers are linked together by a number of contacts: several residues from the N-terminus of the CA-CTD, together with helix 9, form a twofold interface, whereas helices 10 and 11 are key structural elements at the threefold interface (Fig. 2B, right). The structure of the mature lattice is stabilized by abundantly present water molecules that modulate interactions between CA monomers (Gres *et al.*^2015^). Small structural movements between two domains of the CA monomer, as well as between CA-CTDs at the two- and three-fold axis inter-hexameric interfaces in the mature capsid, provide twists and tilts necessary for variable curvature of the conical structure (Mattei *et al.*2016a). In contrast to hexamers, cryo-ET analysis of CA pentamers in the mature cores in intact virions (Mattei *et al.*2016a) revealed significant differences in the arrangement of CA protomers compared to the previously described structure of cross-linked pentamers (Pornillos *et al.*^2011^). Pentamers were found at sites of high curvature (Mattei *et al.*2016a) suggesting that either angle of curvature determines where the 12 pentamers are placed, or pentamer positions determine the angle of curvature of the mature lattice. These cryo-ET results also revealed that pentamers expose different amino acid residues on the outer surface of the capsid relative to hexamers (Mattei *et al.*2016a); these findings have potential implications for the interaction of capsids with host factors post-entry (see below).

### CA-Targeted Inhibitors of HIV-1 Assembly and Maturation

The diverse role of CA in HIV-1 assembly and maturation suggests that CA could be a good target for therapeutic intervention. Currently, none of the antiretroviral drugs in clinical use inhibit the late stages of the HIV-1 replication cycle by targeting Gag-mediated steps in assembly or maturation. However, a number of CA-targeted inhibitors that block virion assembly and/or maturation have been identified and characterized [reviewed in Tedbury and Freed (2015), Spearman (2016) and Carnes *et al.*2018a)]. Some of CA-binding compounds are briefly described below. One of the most promising groups includes bevirimat (BVM), a betulinic acid-derived compound, and its derivatives, which block the proteolytic release of CA from CA-SP1 (Li *et al.*^2003^; Zhou *et al.*^2004^; Urano *et al.*^2016^). BVM, the first-in-class maturation inhibitor, was suggested to bind inside the six-helix bundle (Schur *et al.*^2016^; Wagner *et al.*^2016^; Purdy *et al.*^2018^), thereby stabilizing its structure and preventing CA-SP1 cleavage. Virions produced in the presence of BVM exhibit aberrant morphology characterized by spherical, acentric cores and an electron-dense layer under the viral membrane (Li *et al.*^2003^) that is a stabilized remnant of the immature Gag lattice (Keller *et al.*^2011^). Although BVM did not proceed beyond phase II clinical trials due to the presence of Gag polymorphisms that reduced compound efficacy in a significant number of patients, development of more potent BVM analogs and other compounds with similar mechanism of action has been proceeding (Nowicka-Sans *et al.*^2016^; Urano *et al.*^2016^). CA-SP1 processing can also be blocked by another small-molecule inhibitor, PF-46396 (Blair *et al.*^2009^; Waki *et al.*^2012^). Although this compound is structurally distinct from BVM, it likely acts via a similar mechanism, involving stabilization of the CA-SP1 six-helix bundle. The propagation of viruses in the presence PF-46396 led to the selection of resistant mutants, including several that also confer resistance to BVM (Waki *et al.*^2012^). These results suggest that PF-46396 and BVM share a similar binding site (Blair *et al.*^2009^; Waki *et al.*^2012^). Selection experiments performed with BVM (Adamson *et al.*^2006^) and PF-46396 (Waki *et al.*^2012^) led to the emergence of resistant viruses that were dependent on the maturation inhibitor for their replication. These maturation inhibitor-dependent mutants off-set the stabilizing effect of maturation inhibitor binding by destabilizing the six-helix bundle. These destabilizing mutants were in turn off-set by acquisition of the SP1-T8I mutation, which, like maturation inhibitor binding, stabilized the six-helix bundle (Waki *et al.*^2012^; Fontana *et al.*^2016^). These studies revealed a subtle balance in the stability of the CA-SP1 bundle; this region of Gag needs to be sufficiently stable to enable assembly of the immature Gag lattice, but also sufficiently conformationally dynamic to allow exposure of the CA-SP1 cleavage site and PR-mediated cleavage at that site.

A set of CA inhibitors that bind to either the CA-NTD or the CA-CTD have also been described. CAP-1 is a small molecule that binds at the base of the CA-NTD and inhibits capsid assembly, most likely by disrupting intersubunit CA-NTD–CA-CTD interactions within the mature hexamer (Tang *et al.*^2003^; Kelly *et al.*^2007^). A relatively high concentration of the compound, ~ 100 μmol/L, is needed to reduce infectivity of produced viral particles by 95% in cell-based assays (Tang *et al.*^2003^). The CAP-1-targeting pocket of the CA-NTD overlaps with the binding site of two other groups of compounds—benzodiazepines (BDs) and benzimidazoles (BMs). Both families inhibit the formation of mature virions, albeit by different mechanisms. While the BD compounds significantly inhibit virion release, and the produced virus particles exhibit morphological defects, the BM inhibitors prevent the formation of mature cores but only modestly affect viral production (Fader *et al.*^2011^; Lemke *et al.*^2012^). One of the developed BM-based compounds, compound 1 (C1), has also been shown to interfere with assembly of the mature capsid; this inhibitor binds to a unique site on the top of the CA-NTD, near the base of the CypA-binding loop (Goudreau *et al.*^2013^; Lemke *et al.*^2013^; Wang *et al.*^2017^). A defect in formation of mature infectious virions was also observed upon treatment of HIV-infected cells with the small molecule PF74, which binding site is located at the intersubunit CA-NTD–CA-CTD interface in the mature hexamer (Blair *et al.*^2010^; Bhattacharya *et al.*^2014^; Price *et al.*^2014^). This compound exhibits a dual antiviral activity, inhibiting the assembly of the mature core and early stages of viral infection. Recently, a compound GS-CA1, that acts similar to PF74 by inhibiting early and late stages of viral replication and occupies the same binding site as PF74, showed very promising data and is being tested in clinical development studies (Perrier *et al.*^2017^; Tse *et al.*^2017^). Another CA-targeted compound, a 12-mer α-helical peptide CAI (capsid assembly inhibitor), binds to the hydrophobic cavity formed by CA helices 8, 9 and 11 located in the CA-CTD, thus altering the CA-CTD dimer interface, and inhibits immature- and mature-like particle assembly *in vitro* (Sticht *et al.*^2005^; Ternois *et al.*^2005^). Although CAI was effective in *in vitro* experiments it failed to demonstrate inhibitory activity in cell-based assays due to a low membrane permeability. To enhance its cell permeability, CAI was modified by hydrocarbon stapling (Bhattacharya *et al.*^2008^; Zhang *et al.*^2008^). One of such CAI derivatives, NYAD-1, was able to penetrate into cells and inhibit viral production and immature- and mature-like particle assembly in cell-based systems (Zhang *et al.*^2008^). Another group of CA inhibitors includes modified 2-arylquinazoline compounds that were shown to target the same CA-CTD pocket as CAI *in vitro*, and inhibit viral replication at low micromolar concentrations (Machara *et al.*^2016^). The most likely mechanism of action of these compounds is inhibition of viral particle assembly and formation of the mature core, although additional studies need to be performed to exclude an off-target effect. Given that many of the reported CA-binding inhibitors display weak antiviral activity or exhibit insensitivity to naturally occurring Gag polymorphs, studies on identification and characterization of compounds interfering with structural functions of CA is of great importance to develop and implement novel anti-HIV-1 therapeutic drugs.

## The Roles of CA during Post-Entry and Nuclear Import Events

### “Uncoating”

**Fig. 3:**
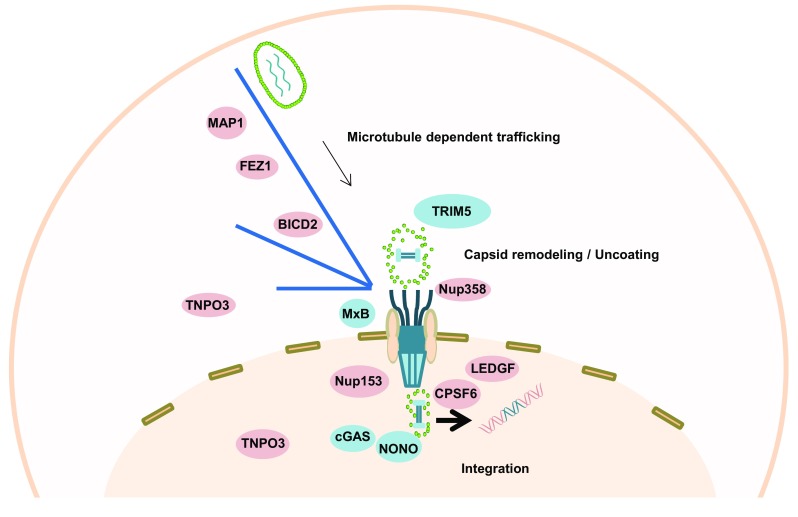
A proposed model for post-entry events of HIV-1 infection mediated by CA-interacting host factors. Microtubule-associated proteins MAP1, kinesin-1 adaptor protein FEZ1, and dynein adaptor protein BICD2 bind to CA protein and facilitate the inward trafficking of HIV-1 reverse transcription complex (RTC) and pre-integration complex (PIC) towards the nuclear membrane. Upon arrival at the nuclear pore complex (NPC), Nup358 mediates docking of RTC/PIC through interacting with CA. Nup153 then mediates PIC translocation through the NPC by interacting with CA, and potentially facilitates nuclear PIC (n-PIC) release from the nuclear basket in concert with CPSF6. Afterwards, CPSF6, together with LEDGF, associate with the n-PIC to facilitate HIV-1 integration. TNPO3 might indirectly facilitate these processes through mediating proper CPSF6 nuclear localization. Several restriction factors also interfere with these post-entry infection events through interacting with CA. TRIM5 binds to the CA of RTC/PIC causing premature uncoating and inhibits reverse transcription. MxB restricts RTC/PIC nuclear entry by interacting with CA. Nuclear factor NONO binds to the CA on n-PIC and promotes cGAS sensing of HIV viral DNA genome in the nucleus. CA in the incoming viral capsid, RTC, and PIC is indicated in green. Viral nucleic acid (RNA or DNA) is shown inside the CA-containing complexes. Host dependency factors are shown in pink and host restriction factors in blue.

After being released into the cytoplasm, the HIV-1 core is transformed into a viral ribonucleoprotein complex to accommodate reverse transcription of the single-stranded viral RNA genome into double-stranded DNA, and to facilitate integration of the viral genome into the host chromosome (Hu and Hughes 2012). Accordingly, these complexes are referred to as the viral reverse transcription complex (RTC) and pre-integration complex (PIC), respectively. The RTC/PICs are believed to undergo a so-called “uncoating” event, losing some of the CA protein and/or the capsid lattice during reverse transcription and nuclear entry (Ambrose and Aiken 2014) (Fig. 3). But the mechanism of when, where and how the “uncoating” event occurs has long been an important yet highly debated topic. One model proposes that the capsid structure needs to be fully disassembled for the virus to complete reverse transcription and nuclear entry (Campbell and Hope 2015). However, accumulating evidence from different groups has demonstrated that a capsid-derived structure is associated with the viral RTC/PIC complexes and facilitates a number of early infection events in the cytoplasm and, potentially, also in the nucleus.

Discrepancies among different studies may derive from the ill-defined physical features of these complexes. A major complication that challenges the investigation of early post-entry events is that only a minority of the incoming viral particles undergoes productive infection and the large amount of abortive infection events may cause noise/background signals that compromise the final readout. Development of modern imaging strategies that can track replication complexes undergoing productive infection has provided a unique opportunity to observe these rare events *in situ*. Together with complementary methods, modern imaging investigation has led to a series of novel findings supporting the functional presence of CA, or even a capsid-derived structure, during early HIV-1 infection events (Peng *et al.*^2014^; Chin *et al.*^2015^; Stultz *et al.*^2017^; Francis and Melikyan 2018). In the following sections we will summarize evidence that supports “complete uncoating” and evidence that supports the functional presence of CA during early infection events. We will then try to reconcile these observations and propose a working model that will evolve with future in-depth investigation.

#### Evidence Supporting “Complete Uncoating” and Proposed Roles for Uncoating

In the early reports of characterizing components of RTC/PICs, the viral replication complexes were isolated from the cytoplasm of infected cells. Cells were lysed followed by fractionation over sucrose gradients by ultracentrifugation (Miller *et al.*^1997^; Fassati and Goff 2001). The fraction that contained detectable HIV-1 DNA was considered to contain RTCs/PICs and was analyzed for the presence of viral proteins. In these efforts, MA, RT and IN were readily detectable but little or no CA was detected (Miller *et al.*^1997^; Fassati and Goff 2001). In contrast, characterization of RTC/PIC components of murine leukemia virus (MLV) showed clear CA association (Fassati and Goff 1999). Accordingly, it was proposed that the HIV-1 CA protein was most likely lost during reverse transcription. It is currently believed that the capsid/capsid-derived structure undergoes dynamic structural remodeling during these early events resulting in metastable complexes (Campbell and Hope 2015; Yamashita and Engelman 2017). It is possible that the remodeled structure is not stable enough to withstand the *in vitro* purification steps employed in these pioneering studies. Recently, some studies reported that CA becomes undetectable within 60 min after virus entry on the majority of the intracellular viral complexes, which is consistent with the proposal that uncoating precedes the completion of reverse transcription (Hulme *et al.*^2011^; Xu *et al.*^2013^). In these studies, investigators made use of the earlier finding that a TRIM-CypA fusion protein binds to the CA protein on incoming capsids and restricts HIV-1 infection (Perez-Caballero *et al.*^2005^; Yap *et al.*^2006^). Cyclosporine A (CsA) prevents the binding between TRIM-CypA and CA, thereby reversing the inhibitory activity of TRIM-CypA. The timing of CsA removal (CsA wash-out) post-infection thus provides a measure of the kinetics of uncoating, enabling the analysis of the relationship between reverse transcription and uncoating. Based on this assay, the authors proposed that reverse transcription accelerates capsid dissociation (Hulme *et al.*^2011^). However, two other studies showed that blocking RT, either by using a RT inhibitor or by introducing mutations in RT, did not affect capsid dissociation *in vitro* or *in vivo* (Kutluay *et al.*^2013^; Xu *et al.*^2013^). Taken together, the potential relationship between “uncoating” and reverse transcription still awaits further characterization and the potential reason why CA was not detected on the RTCs/PICs in these studies will be discussed in the following section.

#### Evidence Supporting the Functional Presence of CA on RTC/PIC

Lentiviruses are unique among retroviruses in their ability to infect non-dividing cells. Being able to integrate their newly synthesized DNA genomes in the host cell chromatin without the dissolution of the nuclear envelope (NE) that occurs during mitosis implies that the lentiviral PIC must be able to cross the intact NE. Initial studies suggested that MA and Vpr were responsible for HIV-1 nuclear import (Bukrinsky *et al.*^1993^; von Schwedler *et al.*^1994^; Gallay *et al.*1995a, b); however, these results were contested (Freed and Martin 1994; Freed *et al.*^1995^, ^1997^). In recent years, a number of studies have shown the important functionality of CA and/or capsid structure during the early infection events of the RTC/PIC pathway. HIV-1 CA was first indicated to be important for early infection events by studies reporting that CA is the determinant for infection of non-dividing cells, as replacing HIV-1 CA with MLV CA in Gag chimeras impaired viral nuclear entry (Yamashita and Emerman 2004; Yamashita *et al.*^2007^). Consistent with this discovery, it has been demonstrated that host factors, including Transportin 3 (TNPO3), Nucleoporin 153 (Nup153) and RAN binding protein 2 (RanBP2), facilitate HIV-1 nuclear entry in a CA-dependent manner (Matreyek and Engelman 2011; Zhou *et al.*^2011^). Imaging-based investigations provided direct evidence of the functional presence of CA on the RTC/PIC in infected cells. In 2002, a study combining fluorescent imaging with electron microscopy to visualize RTCs/PICs in infected cells observed that around 67% of the complexes contained CA proteins (McDonald *et al.*^2002^). However, this pioneering effort could not ascertain whether the association of CA with the RTC/PIC is functionally relevant. In 2014, a robust EdU labeling strategy was used to identify RTC/PICs that have undergone reverse transcription. This study found that, in contrast to the previous study, nearly all the cytoplasmic RTCs/PICs contained detectable CA signal arguing for the functional presence and relevance of CA in these complexes (Peng *et al.*^2014^). It is not clear why these two studies reported different levels of CA association. It should be noted that a polyclonal CA antibody was used to detect CA in the Peng *et al.* study, while a monoclonal antibody was used in the McDonald *et al.* study. The polyclonal CA antibody provides the advantage that it can recognize multiple CA epitopes while the single epitope recognized by the CA monoclonal antibody may be shielded by conformational changes and/or associated host factors especially in the later stage of the RTC/PIC pathway. It is possible that different antibodies used in these two studies could partially explain the inconsistency. This may also explain why other previous studies that used CA monoclonal antibodies did not detect CA on viral complexes after 1 h of infection (McDonald *et al.*^2002^; Xu *et al.*^2013^). It should also be noted that CA association with cytoplasmic RTCs/PICs appears to be cell type dependent, as less than 50% of cytoplasmic RTCs were CA positive in infected monocyte-derived macrophage (MDM), a natural HIV-1 target cell (Peng *et al.*^2014^). Whether this is due to the presence of host restriction factors in MDM that cause premature HIV-1 uncoating needs further investigation. Nevertheless, the functional presence of CA on cytoplasmic RTCs has been confirmed by a number of studies using a variety of labeling strategies (Chin *et al.*^2015^; Francis *et al.*^2016^; Stultz *et al.*^2017^; Francis and Melikyan 2018).

The inconsistencies regarding the presence and functional relevance of CA in the RTC/PIC pathway are likely explained by the use of different methods, materials, and systems in the above-mentioned studies. However, accumulating evidence supports the functional presence of CA throughout the HIV-1 early infection events including cytoplasmic trafficking, NE docking and nuclear import. In the following sections, the roles of CA and interacting host factors during these different stages of HIV-1 early infection will be discussed in further detail.

### CA-Mediated Cytoplasmic Trafficking

The cytoplasm is a dense environment with a high concentration of cellular proteins and organelles. Cargos with a molecular mass larger than 500 kDa cannot diffuse freely and the mega-dalton viral replication complexes are clearly too large for passive diffusion. For this reason, viruses utilize the cytoskeletal network to achieve directed movement (Walsh and Naghavi 2019). HIV-1 has been reported to traffic towards the nucleus along microtubules in a CA-dependent manner, and a number of host factors have been implicated in this interaction (Fig. 3).

#### MAP1A and MAP1S

As the microtubule network has been suggested to provide a path for the movement of HIV-1 replication complexes towards the nucleus, the association between RTCs/PICs and microtubules is likely to be important for HIV-1 trafficking. A yeast two-hybrid screen identified the microtubule-associated proteins MAP1A and MAP1S as interaction partners for HIV-1 CA. Depletion of these two MAP1 proteins reduced HIV-1 capsid association with microtubules, impaired HIV-1 trafficking towards the nucleus and resulted in reduced infectivity. Taken together, these results suggest that MAP1 proteins tether incoming viral capsids to the microtubule network through binding to CA and promote HIV-1 trafficking towards the nucleus (Fernandez *et al.*^2015^).

#### FEZ1 and Kinesin-1

Adaptor and motor proteins mediate cargo translocation along microtubules. Identification of adaptor and motor proteins that mediate HIV-1 trafficking would be important for understanding the mechanism of HIV-1 trafficking along microtubules. Malikov *et al.* identified Fasciculation And Elongation Protein Zeta 1 (FEZ1) as a kinesin-1 adaptor protein that binds CA during HIV-1 infection (Malikov *et al.*^2015^). FEZ1 depletion resulted in viral particles exhibiting bi-directional movement without net trafficking to the nucleus. The interaction between FEZ1 and kinesin-1 was shown to be important for the ability of FEZ1 to promote HIV-1 infection, suggesting that FEZ1 mediates kinesin-1-dependent HIV-1 inward trafficking along microtubules. Interestingly, kinesins are motor proteins involved in plus-end-directed movement which, intuitively speaking, would not facilitate HIV-1 inward trafficking. In addition, the authors found that both dynein and kinesin-1 motors are required for HIV-1 trafficking towards the nucleus. As described below, the mechanism by which HIV-1 employs opposing motors to achieve microtubule-dependent inward trafficking began to be clarified by recent discoveries of dynein adaptor’s participation in this process.

#### BICD2

Bicaudal D2 (BICD2) is a dynein adaptor protein that was recently found in two studies to facilitate HIV-1 trafficking in the cytoplasm (Dharan *et al.*^2017^; Carnes *et al.*2018b). Depletion of BICD2 did not affect reverse transcription but lead to significantly reduced nuclear entry (Dharan *et al.*^2017^; Carnes *et al.*2018b). Using live-cell imaging, Dharan *et al.* further revealed that BICD2 depletion reduced the speed and directed transport of cytoplasmic HIV-1 capsids, resulting in a nuclear entry defect (Dharan *et al.*^2017^). BICD2 was found to interact with intracellular HIV-1 capsids and *in vitro* CA-NC complexes. The CC3 domain of BICD2 was shown to be critical for this interaction (Dharan *et al.*^2017^; Carnes *et al.*2018b). Depletion of BICD2 resulted in accumulation of viral replication complexes in the cytoplasm, which triggered stronger interferon-I (IFN-I) responses in infected differentiated THP-1 macrophages (Dharan *et al.*^2017^). Together, these two studies established the role of dynein adaptor BICD2 in mediating HIV-1 cytoplasmic trafficking towards the nucleus. Thus, both kinesin and dynein adaptor proteins contribute to HIV-1 inward movement. Whether and how the two adaptor proteins (FEZ1 and BICD2) function in concert to mediate HIV-1 inward trafficking is an interesting question for future studies.

It should also be noted that there are studies suggesting that microtubules are dispensable for RTC/PIC cytoplasmic trafficking (Vinay Pathak, personal communication). Indeed, microtubule-independent cytoplasmic trafficking was observed in live-cell imaging (McDonald *et al.*^2002^) and disruption of the microtubule network by nocodazole treatment inhibited HIV-1 infection by only approximately twofold (Bukrinskaya *et al.*^1998^). Further investigation is needed to establish the role of microtubules during RTC/PIC trafficking.

### CA-Mediated NE Docking

Entry through the nuclear pore complex (NPC) is a major pathway for HIV-1 nuclear import, particularly during infection of non-dividing cells such as terminally differentiated macrophages. After inward cytoplasmic trafficking, HIV-1 RTC/PICs dock at the cytoplasmic side of the NPC to initiate nuclear import. Current studies suggest that Nucleoporin 358 (Nup358)/RanBP2 (hereafter referred to as Nup358) is an important nucleoporin mediating RTC/PIC docking at the NPC (Di Nunzio *et al.*^2012^; Burdick *et al.*^2017^) (Fig. 3). Nup358 is a component of the cytoplasmic filament of the NPC (Walther *et al.*^2002^) and promotes nuclear import in a cargo- and transport receptor-specific manner (Walde *et al.*^2012^). The role of Nup358 in HIV-1 replication was first identified in genome-wide screens for host factors required for HIV-1 infection (so-called HIV-1 dependency factors) (Brass *et al.*^2008^; Konig *et al.*^2008^). Depletion of Nup358 impaired HIV-1 nuclear entry as revealed by a decrease in the accumulation of 2-LTR circles (Di Nunzio *et al.*^2012^), often used as a measure of productive nuclear import. Nup358 interacts with *in vitro*-assembled HIV-1 CA-NC complexes (Di Nunzio *et al.*^2012^), which serve as a surrogate for mature capsids (Ganser *et al.*^1999^), and directly associates with intracellular viral replication complexes (Dharan *et al.*^2016^; Burdick *et al.*^2017^). The N74D and P90A mutations in CA impair this association and these viral mutants do not rely on Nup358 for nuclear entry (Dharan *et al.*^2016^). Quantitative microscopy further revealed that depletion of Nup358 reduced the number of HIV-1 replication complexes stably associated with the NE (Burdick *et al.*^2017^). Taken together, these studies strongly suggested that Nup358 determines HIV-1 NE docking in a CA-dependent manner, potentially through direct CA–Nup358 interaction.

Interestingly, Nup358 was also reported to be translocated into the cytoplasm in a CA-and kinesin family member 5B (KIF5B)-dependent manner during HIV-1 infection and this cytoplasmic translocation is suggested to be important for HIV-1 nuclear entry (Dharan *et al.*^2016^). It has been proposed that cytoplasmic translocation of Nup358 may disrupt the NPC and/or promote HIV-1 uncoating and thus indirectly facilitate viral nuclear entry (Dharan *et al.*^2016^).

### CA-Mediated Nuclear Import

In addition to regulating docking at the NE, CA was also reported to mediate PIC transport through the nuclear pore by interacting with several host factors such as Nup153, TNPO3 and the cleavage and polyadenylation specificity factor 6 (CPSF6) (Fig. 3).

#### Nup153

Nup153 is a component of the basket of the nuclear pore complex and plays an essential role in NPC assembly (Vollmer *et al.*^2015^). Nup153 was first identified as a host dependency factor for HIV-1 infection through genome-wide screening (Brass *et al.*^2008^; Konig *et al.*^2008^). Depletion of Nup153 did not affect reverse transcription but impaired HIV-1 nuclear import as revealed by reduced HIV-1 2-LTR circle accumulation and nuclear PICs (Matreyek and Engelman 2011; Di Nunzio *et al.*^2012^, ^2013^). CA was found to determine the Nup153 dependency and the CA mutants N74D and P90A were shown to be largely insensitive to Nup153 depletion (Matreyek and Engelman 2011). Biochemical analysis further revealed that Nup153 directly interacts with *in vitro* assembled HIV-1 CA–NC complexes and CA monomers (Di Nunzio *et al.*^2013^; Matreyek *et al.*^2013^; Buffone *et al.*^2018^). It was therefore proposed that Nup153 facilitates HIV-1 nuclear entry by directly binding to CA molecules on RTCs/PICs. Notably, Nup153 binds CA hexamers with much higher affinity than CA monomers (Price *et al.*^2014^) suggesting that at least some hexameric CA remains intact on RTCs/PICs during transport through the nuclear pore. Depletion of Nup153 was also reported to reduce integration and alter integration site selection (Matreyek and Engelman 2011; Koh *et al.*^2013^). Whether and how these additional functionalities of Nup153 are related to its role in mediating HIV-1 nuclear import will no doubt be the subject of future studies.

#### TNPO3

TNPO3 is a β-karyopherin that transports serine/arginine-rich splicing factors into the nucleus. Like Nup153, TNPO3 was also initially identified as a HIV-1 dependency factor through genome-wide RNA interference screens (Brass *et al.*^2008^). Depletion of TNPO3 did not affect viral reverse transcription but reduced the number of proviruses implying a role during nuclear entry (De Iaco *et al.*^2013^; Fricke *et al.*^2013^). Similarly to Nup153, CA determines TNPO3 dependency and *in vitro* biochemical analysis showed that TNPO3 can bind CA-NC complexes (Krishnan *et al.*^2010^; De Iaco and Luban 2011; Valle-Casuso *et al.*^2012^). Although it was initially proposed that TNPO3 mediates HIV-1 nuclear entry through binding to CA on the RTC/PIC, accumulating evidence supports a role for TNPO3 in facilitating HIV-1 nuclear entry indirectly through regulating the localization of CPSF6 (De Iaco *et al.*^2013^; Fricke *et al.*^2013^; Maertens *et al.*^2014^).

#### CPSF6

CPSF6 is a component of the cleavage factor 1 (CFIm) complex that functions in mRNA polyadenylation. CPSF6 was first identified to be relevant for HIV-1 infection through a cDNA library screen in which a truncated form of CPSF6 was found to inhibit HIV-1 replication at the step of nuclear entry (Lee *et al.*^2010^). It was further revealed that the inhibitory effect of truncated CPSF6 was dependent on a direct CA-CPSF6 interaction. Forced evolution experiments led to the selection of the CA-N74D mutant, which lost CPSF6 binding and escaped the antiviral activity of truncated CPSF6 (Lee *et al.*^2010^, ^2012^). These pioneering discoveries triggered a series of in-depth investigations which suggested that in contrast to truncated CPSF6, which displays cytosolic localization, the intact, full-length CPSF6, which is predominantly nuclear, may function as an HIV dependency factor by facilitating viral nuclear import (Chin *et al.*^2015^). CPSF6 is a serine/arginine-rich protein that is transported into the nucleus by TNPO3. Indeed, depletion of TNPO3 results in significantly higher levels of cytoplasmic CPSF6, which interacts with RTCs/PICs and may consequently impair viral trafficking and/or nuclear entry. This mechanism also explains the discovery that depletion of TNPO3 lead to reduced HIV-1 nuclear entry. Notably, it was shown by two research groups that CPSF6 binds hexameric CA with much higher affinity than monomeric CA (Bhattacharya *et al.*^2014^; Price *et al.*^2014^). This would again suggest that a certain level of hexameric CA remains associated with PICs during and after passing through the nuclear pore. Recent studies reported that the CA-CPSF6 interaction regulates PIC intranuclear localization and directs HIV-1 integration to actively transcribed euchromatin (Sowd *et al.*^2016^; Achuthan *et al.*^2018^). It is possible that the CA-CPSF6 interaction mediates nuclear events beyond nuclear entry and integration; how these events are coordinated by CPSF6 together with other host factors, such as Nup153 and TNPO3, will be interesting questions to follow in the future.

These well-characterized interactions between CA and host proteins unequivocally established the role and functional presence of CA during passage through the nuclear pore. But how this large RTC/PIC passes through the nuclear pore remains enigmatic. The nuclear pore has a central opening of around 40 nm (Bui *et al.*^2013^) which is believed to determine the maximal cargo size (Pante and Kann 2002). While the dimension of the RTC/PIC is currently unknown, it is possible that the diameter may be larger than 40 nm given that the broad end of the HIV-1 capsid was determined to be 56 ± 5 nm (Briggs *et al.*^2006^). Intuitively, it appears difficult to understand how the mega-structure of the RTC/PIC can pass through the nuclear pore. Recent studies reveal that the NPC may undergo dynamic structural re-organization to accommodate translocation of large cargo, especially during viral nuclear entry (Knockenhauer and Schwartz 2016). At the same time, the RTC/PIC likely undergoes structural remodeling and potentially partial uncoating which may result in a complex that fits the opening of the NPC. Furthermore, the fact that CA can bind multiple nucleoporins indicates that the CA protein itself may function as a transportin to facilitate nuclear entry of the “large” RTC/PIC through the nuclear pore. The exact molecular mechanism of RTC/PIC passing through the nuclear pore awaits in-depth investigation and will contribute to the general understanding of transport mechanism of large cargoes through the nuclear pore.

### The Presence of CA on the Nuclear PIC

While the functional relevance of CA during early HIV-1 infection events in the cytoplasm and at the NE has started to become clear, the presence and potential role of CA on the nuclear PIC (n-PIC) is still largely uncharacterized. In 2011, Zhou *et al.* reported detection of nuclear CA in HIV-1 infected cells and further determined the timing of CA nuclear accumulation, implying a role for CA in post-nuclear entry events (Zhou *et al.*^2011^). The presence of nuclear CA was corroborated in a study from Peng *et al.*, in which distinctive CA signals were detected on nearly all n-PICs in infected MDMs (Peng *et al.*^2014^). In that study, viral DNA staining was employed to confirm that the detected n-PICs represented productive replication complexes suggesting that the associated CA may be functionally relevant. The association of CA with nuclear replication complexes was then confirmed by a number of studies from different groups in different infection contexts (Chin *et al.*^2015^; Hulme *et al.*^2015^; Chen *et al.*^2016^; Burdick *et al.*^2017^; Stultz *et al.*^2017^; Francis and Melikyan 2018). Despite the growing consensus that at least some CA remains associated with the PIC after nuclear entry, the role of CA on the n-PIC is not well understood. A study reporting that the CA-CPSF6 interaction contributes to directed HIV-1 integration (Sowd *et al.*^2016^) provides compelling evidence of CA functionality after nuclear entry. A very recent study reported that the host factor NONO binds to HIV CA protein on n-PIC and facilitates cGAS-mediated sensing of HIV DNA in the nucleus (Lahaye *et al.*^2018^). It should be noted that the functional significance of this mechanism is more pronounced for HIV-2 CA than for HIV-1 CA due to stronger binding affinity with NONO (Lahaye *et al.*^2018^). This study not only confirmed the presence of CA on n-PIC but also suggests that the nuclear CA could mediate HIV innate sensing in the nucleus.

### CA-Targeted Restriction Factors

As an integral component of the RTC/PIC, CA not only mediates interactions with host dependency factors to facilitate early infection events but is also the target of several host restriction factors that block the RTC/PIC pathway via different mechanisms (Fig. 3).

#### TRIM5

Tripartite motif-containing protein 5 alpha (TRIM5α) was first identified in an effort to search for species-specific restriction factors that block HIV-1 infection of cells from Old World monkeys (Stremlau *et al.*^2004^). Nonhuman primate TRIM5α proteins, such as rhesus (rh)TRIM5α, inhibit HIV-1 infection by directly binding to the capsid (Wagner *et al.*^2018^). This partially explains why in some cases HIV-1 cannot productively infect cells from nonhuman primates. Binding of rhTRIM5α to capsid causes premature uncoating and inhibits reverse transcription (Black and Aiken 2010; Kutluay *et al.*^2013^). Further analysis showed that the RING domain of the TRIM5 protein is important for restriction, suggesting that ubiquitin ligase activity is involved (Kim *et al.*^2011^; Lienlaf *et al.*^2011^).

#### MxB

Myxovirus resistance protein 2 (MxB/Mx2) (hereafter denoted MxB) is a recently identified HIV-1 restriction factor that is induced by IFNα (Goujon *et al.*^2013^; Kane *et al.*^2013^; Liu *et al.*^2013^). MxB does not inhibit reverse transcription but blocks nuclear entry, as revealed by a reduction in the accumulation of 2-LTR circles. Several studies have established that MxB targets CA to restrict HIV-1 nuclear entry (Busnadiego *et al.*^2014^; Buffone *et al.*^2015^; Schulte *et al.*^2015^). Domain mapping further determined that the N-terminal domain (NTD) of MxB determines restriction against HIV-1 (Goujon *et al.*^2014^). Strikingly, adding the MxB NTD to non-restrictive factors such as MX1 or canine MxB rendered these chimeric proteins restrictive to HIV-1 infection (Busnadiego *et al.*^2014^; Goujon *et al.*^2014^). Interestingly, CA binding is necessary but not sufficient for MxB restriction (Fribourgh *et al.*^2014^; Fricke *et al.*^2014^). A recent study investigated the functional crosstalk between NPC, MxB, CypA and CA and reported that restriction by MxB is largely dependent on CypA and the composition of the NPC (Kane *et al.*^2018^). Accordingly, the mechanism of MxB restriction is proposed to be context dependent in different cell types with varying levels of nucleoporins and CypA (Kane *et al.*^2018^).

## Conclusion

HIV-1 CA mediates a number of processes required for productive HIV-1 infection. Ongoing studies continue to reveal CA regions important for structural integrity, either of immature or mature HIV-1 virions, as well as novel CA interfaces needed for interaction with host cellular cofactors or restriction factors. Given that CA-targeted inhibitors have not been implemented in clinical use so far, novel data on CA functions not only expand our understanding of HIV-1 biology but also provide useful information that could result in the development of novel antiviral therapeutics.
